# Pilot Study of Pesticide Knowledge, Attitudes, and Practices among Pregnant Women in Northern Thailand

**DOI:** 10.3390/ijerph9093365

**Published:** 2012-09-19

**Authors:** Alyson N. Lorenz, Tippawan Prapamontol, Warangkana Narksen, Niphan Srinual, Dana B. Barr, Anne M. Riederer

**Affiliations:** 1 Department of Environmental Health, Rollins School of Public Health, Emory University, Atlanta, GA 30322, USA; Email: dbbarr@emory.edu (D.B.B.); arieder@emory.edu (A.M.R.); 2 Research Institute for Health Sciences, Chiang Mai University, Chiang Mai 50200, Thailand; Email: tprapamontol@rihes.org (T.P.); varangift@hotmail.com (W.N.); nuni21@windowslive.com (N.S.)

**Keywords:** pesticides, prenatal exposure, pregnancy, knowledge, practices, Thailand, agriculture

## Abstract

An estimated 200,000 children born in Thailand each year are at risk of prenatal exposure to pesticides and associated neurodevelopmental outcomes because of their mothers’ agricultural occupations. Children born to non-agricultural workers may also be at risk of exposure from other pathways of maternal pesticide exposure, including exposure through home use, diet, and other environmental media. Pesticide exposure in Thailand has been linked to unsafe practices and beliefs about pesticides. However, limited information exists on pesticide knowledge, attitudes, and practices among pregnant women in Thailand or elsewhere. Obtaining this information is essential to understand the factors associated with prenatal pesticide exposure, identify populations potentially at risk, and ultimately protect pregnant women and their children. We administered surveys to 76 pregnant women in northern Thailand and used multivariable logistic regression to evaluate associations among pesticide-related knowledge, pregnancy trimester, and pesticide use behavior. In this pilot study, lower knowledge score and earliest trimester of pregnancy were marginally (*p *< 0.1) associated with unsafe practices in the home, but not at work. Women who worked in agriculture or applied pesticides before becoming pregnant, or who had a previous child were significantly (*p *< 0.05) more likely to engage in unsafe behaviors in the home during their current pregnancy. We preliminarily conclude that increasing pesticide-related knowledge among pregnant women may help promote safe practices and reduce prenatal exposure. Knowledge-based interventions may be most effective when implemented early in pregnancy and targeted to agricultural workers and other sub-populations at risk of pesticide exposure.

## 1. Introduction

In addition to their agricultural use in crop protection, pesticides are important public health tools that are used to prevent vector-borne disease and to increase food supplies. However, recent research has shown that pesticides may also have negative impacts on public health. Studies have demonstrated acutely toxic effects at high doses, as well as chronic effects at low levels of exposure [1]. Organophosphate (OP), carbamate, pyrethroid, and organochlorine insecticides have been shown to cross the human placenta, exposing developing fetuses [2,3,4]. Prenatal exposure to pesticides is of particular concern due to the demonstrated neurodevelopmental toxicity of certain classes of pesticides (reviewed in [5]). Due to the potential health effects of pesticide exposure, most countries have developed regulations to encourage safe use and control production, import, and export of pesticides. Nonetheless, regulation and enforcement is weaker in some countries than others [6,7]. For example, some pesticides that are banned in certain countries due to their demonstrated health or ecological effects are still used elsewhere [7,8]. In addition, safe practices, such as the use of personal protective equipment and following recommendations on pesticide container labels, are weak or absent in some places [9].

The use of chemical pesticides in Thailand dates back to World War II, when DDT was imported to control the spread of malaria [10]. Since then, their use has expanded to agricultural, industrial, and residential pest control. Most pesticides used in Thailand are imported rather than produced in-country, likely due to the difficulty in obtaining a permit for production from the government [10]. The most recently published statistics showed that over 50,000 tons of active ingredients of pesticides (including insecticides, fungicides, herbicides, and other classes) were imported into Thailand in 2003 [10]. In the same year, 54% of agricultural holdings reported using pesticides, with 73% of holdings in the northern region of the country reporting use [11].

Evidence exists of pesticide-related health effects in Thailand. In 2007, 1,452 pesticide poisoning incidents were reported to the Ministry of Public Health, equivalent to 2.3 per 100,000 population [12,13]. The true number is likely higher, as reported incidents include only those individuals with symptoms severe enough to require medical attention and/or with access to healthcare [12]. In addition, underdiagnosis and underreporting of acute pesticide poisoning are well-recognized issues in developing countries and may also contribute to higher than recorded pesticide poisoning incidents [9]. About 28% of farmers tested by the Ministry of Public Health in 2006 had unsafe levels of cholinesterase depression, a marker of OP and carbamate pesticide exposure [12,14].

In Thailand, 38% of the national workforce was employed in agriculture in 2010 [15]. Although agricultural workers, particularly those involved in pesticide application, are generally considered most at risk of health effects associated with pesticide exposure, the general population can also be exposed through environmental media (e.g., house dust, soil) and consumption of foods with pesticide residues. One study found detectable levels of six different pesticides (dicofol, dieldrin/aldrin, endosulfan, heptachlor/heptachlor epoxide, BHC, and DDT) in domestic water wells in central Thailand [16]. Notably, four of these were banned over 15 years before the study was conducted.

Unsafe practices can lead to measureable health effects in workers exposed to pesticides [17]. Interview surveys of agricultural workers in Thailand linked unsafe pesticide practices such as failing to use personal protective equipment (PPE) and using a higher than recommended concentration of pesticide to decreased serum cholinesterase activity, a marker of OP and carbamate pesticide exposure [12,18]. Further, focus groups and surveys have shown that Thai farmers’ pesticide practices do not always reflect their individual risk beliefs [12,19,20]. In one study, farm workers who believed they were less susceptible to the health effects resulting from pesticide exposure were more likely to have abnormal serum cholinesterase levels than those who believed they were more susceptible [12]. To address unsafe practices and beliefs, researchers recommend educational interventions, which have been shown in several studies to increase knowledge, alter attitudes, and improve pesticide practices in Thailand [21,22].

Researchers in Thailand have found higher levels of organochlorine pesticides and their metabolites in umbilical cord blood than those reported in similar studies from Canada, Australia, and elsewhere [23,24,25]. In addition, Panuwet *et al*. [26] reported higher detection frequencies and median concentrations of OP pesticide metabolites in spot urine samples from children aged 12–13 (n = 207) in Chiang Mai Province, Thailand, than those reported for children aged 11–19 in the United States.

An estimated 200,000 children born in Thailand each year are at risk of prenatal exposure to pesticides resulting from their mothers’ agricultural occupation [27,28]. This number does not take into account other pathways of maternal pesticide exposure, including exposure through home use, diet, and other environmental media, and is thus likely an underestimate. Limited information is available on pesticide knowledge, attitudes, and practices of pregnant agricultural workers and other pregnant women in Thailand and elsewhere [29]. Obtaining this information is essential to understand the factors influencing prenatal pesticide exposures in order to develop interventions that prevent or reduce these exposures. Pesticide exposure is complex and results from a combination of interdependent factors including biological, social, environmental, economic, and political determinants. Knowledge, attitudes, and practices (KAP) surveys help identify knowledge gaps, behavioral patterns, and commonly-held beliefs in order to increase understanding of issues and elucidate targets and themes for interventions that may address any combination of these determinants [30]. KAP surveys focusing on pesticide use have been conducted in several countries including Brazil, Ghana, South Africa, Egypt, and Thailand [21,31,32,33,34]. However, no studies published to date have focused specifically on pregnant women.

We conducted a pilot KAP survey of 76 pregnant women in an agricultural community in Thailand in 2011. Our main objective in conducting the KAP survey was to collect data needed to inform the design of interventions to decrease pesticide exposure in this population. While we plan to conduct an individual-level educational intervention and will focus our analyses and discussion at this level of the ecological model, the information collected through this pilot study could be used to inform interventions at the interpersonal, community, and societal level as well. We hypothesized that pesticide practices would be significantly associated with pesticide knowledge, controlling for demographic characteristics and other relevant covariates. We also hypothesized that pesticide practices would differ by pregnancy trimester, with women in later trimesters using fewer pesticides and adopting behaviors to minimize exposures. 

## 2. Experimental Section

### 2.1. Study Population

This work was a collaboration between researchers from Emory University (Atlanta, GA, USA) and Chiang Mai University (Chiang Mai, Thailand), who are studying neurodevelopmental impacts of prenatal pesticide exposure in the Study of Asian Women and their Offspring’s Development and Environmental Exposures (SAWASDEE) birth cohort. Our target study population included healthy pregnant women, at all stages of pregnancy, using the antenatal care (ANC) clinic at Fang Hospital, Fang District, Chiang Mai Province (Thailand). According to the World Bank, 99% of pregnant women in Thailand received prenatal care in 2009 [35]. Chiang Mai Province was chosen based on previous work showing pesticide exposure in women and children in this area [25,36,37,38,39]. Study nurses enrolled 76 ANC patients during prenatal visits to Fang Hospital in January and February 2011. Participants were volunteers who provided informed written consent. Participation was limited to Thai nationals or foreigners (e.g., Burmese migrant workers) with health insurance cards who had resided in Fang District for at least nine months before enrollment. Guidance and oversight for this work was provided by the human subjects research committees of Emory and Chiang Mai Universities.

### 2.2. Questionnaire Design and Administration

We developed our KAP questionnaire based on previous work conducted by others [31,40,41,42] as well as new questions we designed to collect pregnancy-specific pesticide information. Pesticide knowledge questions collected information on pesticide training, as well as knowledge about exposure routes, populations at risk of exposure, acute and chronic health effects, toxicity symptoms, and effective methods for preventing exposure. Pesticide attitudes questions collected information on beliefs about responsibility for safe use, susceptibility to health effects, pesticide effectiveness, and reasons for pesticide use. Safe practices questions addressed occupational pesticide use, home pesticide use, PPE use, and use of other safety precautions during and after pesticide spraying. Questions regarding pesticide practices were asked prior to pesticide knowledge and attitude questions to avoid biased answers that may result from reflection on pesticide hazards and risks. 

We translated the questionnaire into Thai (and back-translated it to English), pre-tested it with co-workers at Chiang Mai University, and pilot tested it with seven ANC patients during July 2010. Feedback from the pre- and pilot-testing was incorporated into the final questionnaire design. 

The full questionnaire in English is presented in the Supplementary Material. Questionnaires were administered via face-to-face interview in Thai language by two study nurses trained in basic interview techniques. Questionnaire responses were checked by verifying the electronic database against the original paper surveys to ensure accuracy.

### 2.3. KAP Scoring

We calculated knowledge, attitudes, and practices scores using previously published methods, where available. This resulted in seven scores—one measuring knowledge, four measuring attitudes, and two measuring practices. The scores generally did not follow normal or log-normal distributions, thus were dichotomized at the median for the majority of analyses. We calculated a continuous knowledge score that measured the percent of questions answered correctly. This continuous knowledge score was used in the majority of analyses. We also categorized knowledge scores greater than the median as a high degree of knowledge, and those below the median as a low degree of knowledge, using a method modified from Dasgupta *et al.* [43]. Following McCormack *et al.* [44], we considered “don’t know” responses incorrect.

We calculated four separate attitudes scores, including two ‘susceptibility’ attitudes scores: one ranging from 0–4 measuring attitudes about personal susceptibility to the health effects of pesticides, and the other ranging from 0–8 measuring the participant’s attitudes about her child’s susceptibility to the health effects of pesticides. For these scores, the highest values in the ranges indicated the strongest belief in susceptibility to health effects from pesticides, while a score of 0 indicated the weakest belief. Following Sam *et al*. [40], we tabulated a third attitude score, measuring the extent to which a participant believed she had a personal responsibility for the safe use of pesticides. This score ranged from 0–12, with higher values indicating a stronger acceptance of personal responsibility for safe use. We dichotomized these three attitude scores at the maximum score because approximately 50% of participants scored at the maximum on each. We calculated a fourth ‘pesticide usefulness’ attitude score to indicate the degree of the participant’s belief in the usefulness of pesticides. We calculated this score, based on the number of options participants specified as reasons for using pesticides, only for women who personally applied pesticides at work or at home. We dichotomized this score, which ranged from 0–13, at the mean (4.2) because it was approximately normally distributed.

We measured pesticide practices by tabulating the number of reported “risky behaviors” (defined in Goldman *et al*., [45]), or behaviors known or believed to be associated with pesticide exposure, that the participant reported engaging in, including: improper handwashing, delayed bathing, lack of protective clothing, improper storage of clothing, low frequency of house cleaning, eating fruits and vegetables directly from the field, wearing work shoes into the house, and wearing work clothes into the house [46,47,48,49,50,51,52]. We added an additional risky behavior to the Goldman *et al*. list [45]—storing pesticides in or around the home. Since not all of our study participants were agricultural workers, we used two separate measures for risky behaviors—those at work (for agricultural workers) and those at home (for all participants). We dichotomized both pesticide practices scores into ‘no risky behaviors’ or ‘some risky behaviors’. Detailed score calculation procedures are presented in the Supplementary Material.

### 2.4. Statistical Analyses

#### 2.4.1. Descriptive Statistics

We conducted statistical analyses using SAS 9.2 (SAS Institute, Cary, NC, USA). We used non-parametric t-tests (‘Wilcoxon’), parametric t-tests (‘t-test’), chi-square tests (‘chi-sq’), and Fisher’s exact tests (‘Fisher’s’) to compare demographic characteristics and KAP scores of participants who reported working in agriculture (since becoming pregnant) *versus *those who did not. We used α = 0.05 as our criterion for statistical significance and α = 0.1 as indicating a marginally significant or plausible association.

#### 2.4.2. Univariate and Multivariable Logistic Regression Models

We used univariate maximum likelihood logistic regression to evaluate the extent to which the continuous knowledge score and pregnancy trimester (first trimester *versus* second or third trimester) were associated with risky behaviors (‘some’ *versus *‘none’) at work and at home. We then constructed preliminary multivariable logistic regression models containing covariates to further evaluate these associations. In assessing collinearity, interaction, and confounding, we included covariates that were associated with both the ‘primary predictor’ (e.g., knowledge score or pregnancy trimester) and the ‘outcome’ (e.g., risky behaviors at home or risky behaviors at work), identified using non-parametric and parametric t-tests, chi-square tests, and Fisher’s exact tests. Condition indices and variance decomposition proportions (VDPs) were calculated with a collinearity problem identified when at least one condition index was >30 [53]. Covariates with VDPs >0.5 associated with such condition indices were eliminated from the model to ensure stability of maximum likelihood estimates [54]. We assessed interaction using two-way interaction terms between the primary predictor and each covariate with hierarchical backward elimination of interaction terms [55]. Covariates were assessed for confounding by comparing reduced models to the full model with all covariates. When removal of a covariate resulted in an odds ratio (OR) for the primary predictor that differed by >10% from the full model OR, the covariate was considered a confounder and included in the final multivariable model. Participants with missing data for any model variable were excluded from the corresponding analyses. To determine the explanatory power of these models, we used likelihood ratio tests to compare each final model to a corresponding reduced model containing only the intercept. We used Wald chi-square tests (‘Wald chi-sq’) to identify ‘predictors’ that were significantly associated with ‘outcomes’ in each model (Wald chi-sq *p *< 0.05).

#### 2.4.3. Elucidating Targets for Intervention

We identified knowledge gaps using means and proportions to reveal areas where knowledge was least prevalent. We also compared knowledge and behaviors to determine whether specific (rather than general) knowledge of harmful actions and protective strategies led to correspondingly appropriate actions and strategies. To elucidate potential targets for educational interventions, we examined factors associated with inconsistencies in declared knowledge and reported behaviors using t-tests and chi-square tests, comparing participants with inconsistencies *versus *participants without inconsistencies.

**Table 1:** Demographic characteristics of all participants and by occupation.

		All Participants (n = 76) (Mean (SD ^#^)/N (%))	Agricultural Workers (n = 34) (Mean (SD)/N (%))	Non-Agricultural Workers (n = 42) (Mean (SD)/N (%))	*p*-value (Test) for Significant Differences ^^^
**Age (years)**		26.0 (6.8)	26.6 (7.0)	26.1 (6.7)	0.77 (t-test)
**Ethnicity**					0.001 ^*^ (Fisher’s)
	Thai	34 (45%)	8 (24%)	26 (62%)	
	Thai Yai	31 (41%)	20 (59%)	11 (26%)	
	Burmese	2 (3%)	2 (6%)	0 (0%)	
	Chinese	2 (3%)	0 (0%)	2 (5%)	
	Other	7 (9%)	4 (12%)	3 (7%)	
**Born in Thailand**		46 (61%)	14 (41%)	32 (76%)	0.002 ^*^ (chi-sq)
**Highest level of education achieved**					<0.001 ^*^ (Fisher’s)
	None, never attended school	33 (43%)	23 (68%)	10 (24%)	
	Primary school	12 (16%)	5 (15%)	7 (17%)	
	Junior high school	10 (13%)	1 (3%)	9 (21%)	
	High school (no diploma)	15 (20%)	5 (15%)	10 (24%)	
	High school diploma or greater	6 (8%)	0 (0%)	6 (14%)	
**Household monthly income ^†^**					0.002 ^*^ (Fisher’s)
	1,500 Baht or less (≤49 USD)	2 (3%)	2 (6%)	0 (0%)	
	1,501 to 3,000 Baht (50–99 USD)	6 (8%)	4 (12%)	2 (5%)	
	3,001 to 6,000 Baht (100–199 USD)	22 (29%)	13 (38%)	9 (21%)	
	6,001 to 9,000 Baht (200–299 USD)	21 (28%)	8 (24%)	13 (31%)	
	9,001 to 12,000 Baht (300–399 USD)	13 (17%)	1 (3%)	12 (29%)	
	12,001 Baht and above (≥400 USD)	6 (8%)	1 (3%)	5 (12%)	
	Don’t know/Not sure	6 (8%)	5 (15%)	1 (2%)	
**Pregnancy trimester**					0.053 (chi-sq)
	1st	21 (28%)	14 (41%)	7 (17%)	
	2nd	25 (33%)	10 (29%)	15 (36%)	
	3rd	30 (39%)	10 (29%)	20 (48%)	
**Number of pregnancies before current pregnancy**					0.62 (chi-sq)
	0	29 (38%)	13 (38%)	16 (38%)	
	1	30 (39%)	15 (44%)	15 (36%)	
	2 or 3	17 (22%)	6 (18%)	11 (26%)	
**Worked since becoming pregnant**		66 (87%)	34 (100%)	32 (76%)	0.002 ^*^ (Fisher’s)
**Worked in agriculture since becoming pregnant**		34 (45%)	34 (100%)	N/A	N/A

^# ^SD = standard deviation;^ ^ ^Tests for differences between agricultural and non-agricultural workers;^* ^Significant result (*p *< 0.05);^ † ^Approximate, based on the average exchange rate during the period of enrollment (1 USD = 30.4566 THB, www.oanda.com).

Where knowledge score and pregnancy trimester were not significantly associated with risky behaviors, multivariable logistic regression models were constructed to identify other factors associated with risky behaviors. A simple backward elimination procedure was implemented, allowing variables other than knowledge or pregnancy trimester to become a part of the final model. The least significant term was eliminated from the model sequentially until all remaining terms were significant (Wald chi-sq *p *< 0.05).

## 3. Results

### 3.1. Descriptive Statistics

Demographic and pesticide use characteristics of the 76 participants are presented in Table 1 and Table 2. The mean age was 26.0 (±6.8) years, and participants were relatively evenly distributed across the first (28%), second (33%), and third (39%) trimesters of pregnancy. Approximately half (45%) of the participants reported working in agriculture since becoming pregnant. Twenty-three (30%) women, all agricultural workers, reported that pesticides were applied at their job. Pesticides were applied in the homes of 39 (51%) participants since they became pregnant, with 21 (28%) personally applying those pesticides. There were no missing data for any of the variables included in the descriptive statistics.

**Table 2:** Pesticide use characteristics of all participants and by occupation.

		All Participants (n = 76) (N (%))	Agricultural Workers (n = 34) (N (%))	Non-Agricultural Workers (n = 42) (N (%))	*p*-value (Test) for Significant Differences ^^^
**Occupational**					
	Personally applied pesticides at work since becoming pregnant	8 (11%)	8 (24%)	0 (0%)	0.005 ^*^ (Fisher’s)
	Had a job where pesticides were applied since becoming pregnant	23 (30%)	23 (68%)	0 (0%)	<0.0001 ^*^ (chi-sq)
	Worked in a job involving potential pesticide exposure before becoming pregnant	46 (61%)	33 (97%)	13 (31%)	<0.0001 ^*^ (chi-sq)
**Residential**					
	Pesticides used in the home since becoming pregnant	39 (51%)	16 (47%)	23 (55%)	0.50 (chi-sq)
	Pesticides used in the home before becoming pregnant	43 (57%) ^#^	16 (47%)	27 (66%) ^#^	0.10 (chi-sq)
	Personally applied pesticides in the home since becoming pregnant	21 (28%)	9 (26%)	12 (29%)	0.84 (chi-sq)
	Personally applied pesticides in the home before becoming pregnant	26 (34%)	10 (29%)	16 (38%)	0.43 (chi-sq)
	Personally applied pesticides on pets since becoming pregnant	15 (20%)	4 (12%)	11 (26%)	0.12 (chi-sq)

^^ ^Tests for differences between agricultural and non-agricultural workers; ^* ^Significant result (*p *< 0.05); ^# ^Data missing for one participant.

Table 3 presents descriptive statistics for the seven KAP scores for agricultural workers, non-agricultural workers, and all participants together. The median overall knowledge score was 0.86 (IQR = 0.10), and knowledge did not differ significantly between agricultural and non-agricultural workers (Wilcoxon *p *= 0.10). However, agricultural workers scored significantly lower in attitudes about responsibility for the safe use of pesticides and significantly higher in the number of risky behaviors at home (Wilcoxon *p *< 0.01). Of the 34 agricultural workers, 16 (47%) reported regularly engaging in at least one risky behavior at work. Among all participants, 55 (72%) regularly engaged in at least one risky behavior at home.

**Table 3:** KAP scores of all participants and by occupation.

		All Participants (n = 76)	Agricultural Workers (n = 34)	Non-Agricultural Workers (n = 42)	*p*-value (Test) for Significant Differences ^^^
**Knowledge Score (0–1)**					
	Mean (SD)	0.84 (0.07)	0.82 (0.07)	0.85 (0.07)	0.10 (Wilcoxon)
	Median (IQR ^#^)	0.86 (0.10)	0.84 (0.10)	0.86 (0.10)	
	N (%) above median (0.86)	41 (54%)	16 (47%)	25 (60%)	0.28 (chi-sq)
**Personal Susceptibility Attitudes Score (0–4)**					
	Mean (SD)	3.4 (1.3)	3.2 (1.5)	3.6 (1.0)	0.44 (Wilcoxon)
	Median (IQR)	4.0 (0.0)	4.0 (1.0)	4.0 (0.0)	
**Child Susceptibility Attitudes Score (0–8)**					
	Mean (SD)	7.0 (1.8)	6.8 (2.0)	7.2 (1.7)	0.60 (Wilcoxon)
	Median (IQR)	8.0 (2.0)	8.0 (3.0)	8.0 (1.0)	
**Responsibility Attitudes Score (0–12)**					
	Mean (SD)	10.4 (2.0)	9.5 (2.3)	11.1 (1.2)	0.001 ^*^ (Wilcoxon)
	Median (IQR)	11.0 (2.0)	10.0 (4.0)	12.0 (2.0)	
**Usefulness Attitudes Score (0–13)**					
	Mean (SD)	5.2 (2.5)	5.4 (2.9)	4.9 (2.0)	0.61 (t-test)
	Median (IQR)	5.0 (4.0)	4.5 (4.0)	5.5 (3.0)	
**Risky Behaviors at Work Score (0–3)**					
	Mean (SD)	N/A	0.56 (0.66)	N/A	N/A
	Median (IQR)	N/A	0.0 (1.0)	N/A	
	N (%) with at least one risky behavior at work	N/A	16 (47%)	N/A	N/A
**Risky Behaviors at Home Score (0–7)**					
	Mean (SD)	1.4 (1.3)	1.9 (1.2)	1.0 (1.2)	0.002 ^*^ (Wilcoxon)
	Median (IQR)	1.0 (2.0)	2.0 (2.0)	1.0 (1.0)	
	N (%) with at least one risky behavior at home	55 (72%)	30 (88%)	25 (60%)	0.005 ^*^ (chi-sq)

^# ^IQR = interquartile range;^ ^ ^Tests for differences between agricultural and non-agricultural workers; ^* ^Significant result (*p *< 0.05).

Higher knowledge was significantly associated with having at least some formal education (chi-sq *p *= 0.03), receiving pesticide training (Fisher’s *p *= 0.03), and believing in personal responsibility for the safe use of pesticides (Wilcoxon *p *= 0.02). Higher knowledge was also marginally associated with being Thai (chi-sq *p *= 0.09). Women in their first trimester were more likely to be agricultural workers (chi-sq *p *= 0.02) and were less educated than women in later trimesters (chi-sq *p *< 0.01).

**Table 4:** Logistic regression model parameters and fit statistics.

Outcome Variable Tested		Odds Ratio	95% Confidence Interval	Parameter Estimate	Standard Error	*p*-value (Wald chi-sq test)	*p*-value (Likelihood Ratio test)
**A. Risky behaviors at work (n = 34) ^^^**							0.20
	Knowledge score	1.1	(0.9, 1.4)	0.13	0.11	0.21	
	Intercept	N/A	N/A	-5.41	4.28	0.21	
**B. Risky behaviors at work (n=34) ^^^**							0.68
	Pregnancy trimester (1st/2nd or 3rd)	0.8	(0.2, 3.0)	-0.29	0.70	0.68	
	Intercept	N/A	N/A	0.00	0.45	1.00	
**C. Risky behaviors at work (n = 34) ^^^**							0.01 ^*^
	Number of risky behaviors at home	2.2	(1.1, 4.5)	0.79	0.36	0.03	
	Intercept	N/A	N/A	-1.62	0.78	0.04	
**D. Risky behaviors at home (n = 76)**							0.09
	Knowledge score	0.9	(0.7, 1.0)	-0.14	0.08	0.10	
	Intercept	N/A	N/A	6.68	3.54	0.06	
**E. Risky behaviors at home (n = 76)**							0.02 ^*^
	Pregnancy trimester (1st/2nd or 3rd)	5.0	(1.1, 23.9)	1.61	0.80	0.04	
	Intercept	N/A	N/A	0.64	0.28	0.02	
**F. Risky behaviors at home (n = 76)**							0.04 ^*^
	Pregnancy trimester (1st/2nd or 3rd)	4.1	(0.8, 20.6)	1.42	0.82	0.08	
	Education (some/none)	0.6	(0.2, 1.8)	-0.54	0.58	0.35	
	Intercept	N/A	N/A	1.02	0.51	0.04	
**G. Risky behaviors at home (n = 76)**							<0.01 ^*^
	Farmwork before pregnant (yes/no)	9.5	(2.2, 41.8)	2.25	0.76	<0.01	
	Pesticides applied before pregnant (yes/no)	12.2	(2.0, 75.1)	2.5	0.93	0.01	
	Previous child (yes/no)	4.1	(1.0, 15.7)	1.4	0.69	0.04	
	Child's susceptibility score (high/low)	5.8	(1.4, 24.7)	0.76	0.74	0.02	
	Intercept	N/A	N/A	-2.6	0.91	<0.01	

^^ ^Agricultural workers only; ^* ^Significant result (p < 0.05).

### 3.2. Univariate and Multivariable Regression Models

Table 4 presents results from the univariate and final multivariable regression models showing associations with risky behaviors at work and home (any *versus* none). There were no missing data in these analyses, though some questions were not applicable to all participants. In univariate tests of our study hypotheses, engaging in risky behaviors at home was marginally associated with the continuous knowledge score (D; Wald chi-square *p *= 0.10) and significantly associated with pregnancy trimester (E; Wald chi-square *p *= 0.04). Neither factor was associated with the number of risky behaviors at work (A, B; Wald chi-square *p *> 0.20).

The final multivariable model describing the association between the continuous knowledge score and risky behaviors at work contained knowledge as the only predictor. The association between knowledge and risky behaviors at work was positive (A; odds ratio (OR) = 1.1) but not statistically significant (*p *= 0.21; 95% confidence interval (CI) 0.9–1.4) and the likelihood ratio test showed low explanatory power (*p *= 0.20). The final model describing the association between the knowledge score and risky behaviors at home also contained knowledge as the sole predictor, with a negative (D; OR = 0.9) and marginally significant association (*p *= 0.10; 95% CI 0.7–1.0). Explanatory power was also marginally significant (likelihood ratio *p *= 0.09*)*.

The univariate association between pregnancy trimester and risky behaviors at work was not statistically or marginally significant so the corresponding multivariable model was not constructed. The final model of the association between pregnancy trimester (first *versus *second/third) and risky behaviors at home included education as a significant confounder. Although pregnancy stage was significantly associated with risky behaviors at home in the univariate model (E; OR = 5.0, 95% CI 1.1–23.9), the association was not statistically significant when education was included (F; OR = 4.1, 95% CI 0.8–20.6). 

### 3.3. Elucidating Targets for Intervention

Knowledge areas with the lowest median scores were pesticide toxicity symptoms and pesticide exposure intake routes (0.78 and 0.80, respectively, out of 1.0). Only 5% of participants indicated that they knew that different pesticides have different health effects. Although nearly all participants (96%) agreed that spraying pesticides in the home could harm their fetuses, 28 (37%) reported using pesticides in the home since becoming pregnant. Similarly, all of the 18 (24%) participants who reported not wearing gloves while using pesticides in the home expressed knowledge that wearing gloves was an effective strategy to prevent pesticide exposure. Women with these inconsistent responses were more likely than those with consistent responses to report using pesticides in the home before becoming pregnant, and more likely to report engaging in other risky behaviors including smoking and allowing agricultural workers to wear shoes into the home after work (chi-sq and Fisher’s *p *< 0.05).

The regression analyses presented in Table 4 identified populations with increased odds of engaging in risky behaviors and pointed to targets for intervention beyond simple knowledge dissemination. For example, we found that agricultural workers who engaged in more risky behaviors at home were significantly more likely to engage in risky behaviors at work (C; OR = 2.2, 95% CI 1.1–4.5). Having a job involving farm work before becoming pregnant, using pesticides in the home before becoming pregnant, having a previous child, and having a strong belief in the child’s susceptibility to pesticides were significantly associated with engaging in risky behaviors at home (G; Wald chi-sq *p *< 0.05).

## 4. Discussion

### 4.1. Pesticide Knowledge, Attitudes, and Practices in the Study Population

In general, study participants demonstrated relatively high levels of knowledge about pesticides, with most answering over 80% of the knowledge questions correctly. Knowledge was higher among our participants than reported in previous studies of agricultural workers in Thailand [12] and other locations such as Bangladesh, Brazil, and Palestine [31,40,56]. This may be due to our inclusion of non-agricultural workers, who had higher levels of education and higher knowledge scores than agricultural workers.

Consistent with previous findings [19], attitudes on personal susceptibility to the health effects of pesticides were not associated with pesticide practices among the women in our study (Fisher’s *p *> 0.20). In our small cohort, beliefs in personal responsibility for the safe use of pesticides were lower among agricultural than non-agricultural workers. The agricultural workers were also more likely to engage in risky behaviors in the home. However, certain behaviors were only considered risky when the participant had a household member who worked in agriculture, which was significantly more common among participants who worked in agriculture themselves (chi-sq *p *< 0.01). Thus, while it is difficult to assess the true association between working in agriculture and engaging in unsafe practices in the home, we preliminarily conclude that the agricultural workers in our study likely have a greater potential for exposure to pesticides in the home due to the significant association with risky behaviors.

Risky behaviors were less common in our study population than in other populations, including pregnant agricultural workers in California [45] as well as other Thai agricultural worker populations [12,18,57]. While the women in our study may truly be less likely to engage in certain risky behaviors, our pilot tests showed that some participants may be unlikely to admit to engaging in risky behaviors due to a desire to please researchers. Other survey-based studies of behavior among populations of Asian women have introduced similar concerns about the potential for reporting or desirability bias [58,59]. Although we attempted to minimize this bias by asking the behavior questions before the knowledge questions, this bias may not be completely removed through a multiple choice survey conducted in a face-to-face interview [60]. Future studies might consider anonymous surveys or open-ended qualitative surveys, interviews, and focus groups to further minimize this bias (see Flocks *et al*. [29]).

### 4.2. Knowledge and Pregnancy Trimester: Associations with Pesticide Practices

In this study, risky behaviors were used as a measure of potential pesticide exposure during pregnancy to help identify women and children at greater risk of pesticide exposure and reveal potential targets for interventions. Higher knowledge was marginally associated with decreased odds of engaging in risky behaviors at home. On average, the odds of engaging in risky behaviors at home decreased by approximately 10% for every additional knowledge question answered correctly, preliminarily indicating that interventions designed to increase knowledge among pregnant women from all backgrounds could be effective at reducing exposures in the home. However, this association did not meet our criterion for statistical significance, thus a study with a larger sample size would be needed to confirm or reject the relationship. Knowledge was not significantly associated with risky behaviors at work in our study, perhaps also due to small sample size (n = 34 agricultural workers).

As we hypothesized, women in later stages of pregnancy (e.g., second or third trimesters) were significantly less likely to engage in risky behaviors at home. In addition, more women had worked in a job involving potential pesticide exposure, personally applied pesticides, or had pesticides applied in their home before becoming pregnant than after becoming pregnant. These observations may indicate that women alter their pesticide use behaviors when they become pregnant as well as when they advance to later stages of pregnancy. However, when we included education as a covariate in our models, the association between pregnancy trimester and risky behaviors at home was only marginally significant. In our small sample population, first trimester participants were less likely to be educated than participants in their second or third trimesters. Pregnancy trimester was not associated with risky behaviors at work, potentially due to small sample size.

### 4.3. Targets for Intervention

The knowledge gaps we identified in this pilot study could be used to design knowledge-based interventions aimed at pregnant women in Thailand. However, these types of interventions may not be sufficient to reduce or eliminate prenatal pesticide exposures. For example, some of the women we surveyed reported knowledge of risky behaviors but engaged in them nonetheless. These women reported other unsafe practices and their previous pesticide use habits did not appear to be influenced by their becoming pregnant. These important behavioral observations should be considered when planning interventions in this or similar populations.

In addition, regression modeling showed that neither knowledge nor pregnancy trimester alone were significantly associated with risky behaviors, but that other factors were involved. The number of risky behaviors at home was significantly associated with increased odds of engaging in risky behaviors at work. Specifically, the odds of engaging in risky behaviors at work increased two-fold for each risky behavior the participants reported at home. We preliminarily interpret this to mean that interventions designed to decrease the number of risky behaviors at home may be effective in decreasing risky behaviors at work as well. In addition, risky behaviors at home might serve as a proxy for risky behaviors at work in future studies. Behaviors at home can be assessed among all pregnant women, in contrast to behaviors at work, which typically only apply to agricultural workers.

Four other covariates were significantly associated with increased odds of engaging in risky behaviors at home in our study. These observations may help future researchers prioritize sub-groups for educational interventions. Agricultural workers were more likely to report risky behaviors at home, so the additional finding that having an agricultural job before becoming pregnant was significantly associated with risky behaviors in the home was not surprising. However, it helps underscore the importance of identifying agricultural workers as a group at elevated risk of pesticide exposure during pregnancy. Similarly, we observed that participants who reported using pesticides in the home before becoming pregnant were more likely to report unsafe pesticide use behaviors while pregnant. In addition, women with a previous child were significantly more likely to engage in risky behaviors at home during the current pregnancy. This is consistent with previous findings that within certain populations in the United States, women were more likely to engage in potentially harmful behaviors such as use of tobacco, lower utilization of prenatal care, and failure to meet diet quality and nutritional recommendations during later pregnancies than during their first [61,62]. Thus, women who already have children may need reminders that safe pesticide practices during pregnancy are necessary to protect their developing fetus. Although the predictors identified in backward elimination (having a job involving farm work before becoming pregnant, using pesticides in the home before becoming pregnant, having a previous child, and having a strong belief in the child’s susceptibility to pesticides) can help identify populations at risk of exposure, these factors are not preventable through interventions implemented during pregnancy. Thus, our pilot study shows that pesticide knowledge may remain an important target for prevention activities.

### 4.4. Study Limitations

Our questionnaire was based on previously published work, pre-tested, pilot tested, and edited to ensure accurate translation, coherence, and relevance. However, we did not conduct a full-scale validation study, such as in Sam *et al. *[40]. Further, knowledge may be better measured using open-ended questions, where participants are asked to provide information freely, without being influenced by potentially leading questions or a restricted number of choices. Additionally, we did not receive any critical feedback during pilot testing, so some women in our study may have wanted to please the researchers instead of answer questions accurately. Such a reporting bias may partially explain why safe practices were more prevalent in our study than in previous findings.

Our study is also limited due to small sample size (n = 76). A larger number of participants would likely be needed to detect true associations among the variables of interest within smaller subgroups such as agricultural workers or women who personally applied pesticides. Further, our primary ‘outcomes’ of interest were not direct measures of exposure, but proxy questionnaire responses. Although there is evidence that unsafe pesticide practices are associated with increased exposure in Thai populations [12,18], this association has not been studied among pregnant women in Thailand or their children. While it is practical to assume that risky behaviors lead to increased exposures in this population, actual exposure measurements would be needed to validate the assumption.

Finally, behavior change interventions may have only modest impact without changes to national and international pesticide policy. The context of pesticide and agricultural policy in Thailand is unique and challenging, with weak enforcement of existing regulations and the absence of a uniform system for pesticide management [63]. While addressing the issue of prenatal pesticide exposure at the community and societal level is outside the scope of this study, these areas offer another opportunity for intervention and the results of our KAP survey in this understudied population may be useful in such efforts.

## 5. Conclusions and Recommendations

Despite these limitations, we found plausible associations between pesticide knowledge and stage of pregnancy and risky pesticide behaviors in this study population. We make the preliminary recommendation that individual-level interventions designed to reduce prenatal pesticide exposure in this and similar populations should focus on educating women about the hazards of risky pesticide practices in the home, since this can benefit all pregnant women, not just agricultural workers. Knowledge-based interventions might also target the first trimester of pregnancy, since evidence from our small sample indicates that women may engage in more risky behaviors during early pregnancy than later in pregnancy. When resources are limited, interventions might be targeted specifically to agricultural workers and women who already have children, sub-groups we identified as more likely to engage in unsafe pesticide practices during pregnancy. However, we also recommend further research to validate these conclusions in different and larger populations. In sum, although the women in our study were relatively knowledgeable about pesticides, many still reported engaging in risky behaviors, illustrating the urgent need for interventions in this and similar populations of pregnant women worldwide.

## Supplementary Files

Supplementary File 1: PDF-Document (PDF, 721 KB)
