# Supplementary material for: Pilot Study of Pesticide Knowledge, Attitudes, and Practices among Pregnant Women in Northern Thailand
Source: Int J Environ Res Public Health. 2012 Sep 19;9(9):3365–83. doi: 10.3390/ijerph9093365 (PMC3499875; doi:10.3390/ijerph9093365)
Supplement: Supplementary File 1: — PDF-Document (PDF, 721 KB) [file ijerph-09-03365-s001.pdf]

Chiang Mai Birth Cohort – Pilot Study
KAP QUESTIONNAIRE
(Interview immediately after enrollment)

Hello, my name is ______________. I want to start by thanking you for your help with this survey. I want to let you know that all of your answers to these questions are completely confidential. If you feel uncomfortable answering any of these questions, please let me know. We would appreciate your being as honest as possible in your answers. Do you have any questions before we begin? Thank you for helping us with this important project!

INTERVIEWER USE ONLY

Interviewer initials ___________ □□□

Interview date □□/□□/□□□□
Day / Month / Year

Interview start time □□:□□

Language of interview
- Thai..01
- Thai Yai..02
- Burmese..03
- Other (SPECIFY) __________________...........04

Patient's due date □□/□□/□□□□
Day / Month / Year

1. What month of pregnancy are you in? ____ MONTH OF PREGNANCY

A. Occupational Information/Physical Exertion

Now, I would like to ask you some questions about any jobs you may have held since you became pregnant.

1. Have you worked since you became pregnant, since your last menstrual period?

Yes 01
No…….(**NEXT SECTION**) 02
NR 999

		A. Job 1 or most recent job
8.	Since you became pregnant, at this job have you done any agricultural work? (including fertilizer handling and application, agricultural pesticide handling, equipment or tractor operation, foreman of agricultural work, farm field work, packing shed work, nursery or greenhouse work, or waxing fruits)	YES...............01<br>NO…..............02
11	During this pregnancy, at this job did you do…<br><br>i. Golf course or other landscape maintenance? ....<br><br>ii. Control for termites or other pests in homes or buildings? ......<br><br>iii. Work in a cannery or food processing plant where you handle fruits or vegetables?	 <br>YES ....01<br>NO ………….02<br><br>YES ....01<br>NO ………….02<br><br>YES ....01<br>NO ………….02
12	Since you became pregnant, do/did you apply pesticides or insecticides at this job?	YES…………....01<br>NO……………...02
16	Since you became pregnant, are/were pesticides or insecticides used at this job?<br>**(CODE 777 IF NOT WORKING)**	YES………....01<br>NO(**GOTO18**)02<br>NA(**GOTO18**)NA

		A. Job 1 or most recent job
18	During this pregnancy, have you usually worn at this job…	
	i. Long-sleeved shirt?................	YES...............01<br>NO…..............02
	ii. Cotton gloves?	YES...............01<br>NO.................02
	iii. Hat or something covering your head?	YES…...........01<br>NO.................02
	iv. Scarf/handkerchief to cover your face?	YES...............01<br>NO.................02
	v. Rubber boots?........................	YES...............01<br>NO.................02
20	During this pregnancy, when you are/were at this job, do/did you wash your hands before eating or smoking?	ALWAYS........01<br>USUALLY......02<br>SOMETIMES.03<br>NEVER..........04
21	During the time you worked at this job, did you usually bathe or shower...?	
	i. Daily, before work	YES...............01<br>NO…..............02
	ii. Daily, immediately after work at work place	YES...............01<br>NO…..............02
	iii. Daily, immediately after arriving home from work	YES...............01<br>NO…..............02
	iv. Daily, more than 1 hour after arriving home from work	YES...............01<br>NO…..............02
	v. Several times a week	YES...............01<br>NO…..............02
	vi. Once a week or less often	YES...............01<br>NO…..............02

30.	In the year before you became pregnant, have you done…<br><br>A. Farm field work?...................................	 <br>YES............. 01<br>NO.................02
	B. Packing, canning, or food processing where you handled fruits, vegetables, or flowers?..	YES............. 01<br>NO.................02
	C. Nursery or greenhouse work?.............	YES............. 01<br>NO.................02
	D. Golf course or landscape maintenance?...	YES............. 01<br>NO.................02

B. Housing Characteristics

	A…since you became pregnant?	B...in the year before you became pregnant?
10. How often have your fruits and vegetables been washed before you ate them…	Always…………………………..01<br>Usually………………………..02<br>Sometimes……………………03<br>Almost never………………....04<br>DK…………………………...888<br>NR…………………………...999	Always…………………………..01<br>Usually………………………..02<br>Sometimes……………………03<br>Almost never………………....04<br>DK…………………………...888<br>NR…………………………...999
11. Have you eaten fruits and vegetables that came directly from the fields… (Do not include those from your home garden)	Yes…………………………….01<br>No……………………………..02<br>DK…………………………...888<br>NR…………………………...999	Yes…………………………….01<br>No……………………………..02<br>DK…………………………...888<br>NR…………………………...999

C. House Cleaning

2. How often do you clean your home?	Daily or more often .... 01<br>A few times a week .... 02<br>Once a week .... 03<br>Once every couple of weeks .... 04<br>Once a month or less often .... 05<br>DK .... 888<br>NR 999

D. Pesticide Use

I would like to ask you some questions about pesticides that have been used in and around any of the homes you have lived in since you became pregnant. Pesticides can come in sprays, bombs, poison pellets or bait, powder, chalk, roach motels, traps, or ant stakes.

4. Since you became pregnant, have pesticides or insecticides been used around any of your homes to kill pests?	YES .... 01<br>NO… .... 02<br>DK 888
4C. Were these pesticides used inside or outside your home?	INSIDE .... 01<br>OUTSIDE…................. 02<br>BOTH .... 03<br>DK 888

4E. How often have these pesticides been applied?

Daily 01
Weekly… 02
1 to 3 times per month 03
<1 time per month 04
DK 888

4F. Did you personally apply any of these pesticides?

YES 01
NO 02
DK 888

5. **(SKIP IF SHE DID NOT PERSONALLY APPLY PESTICIDE)**
When you applied the pesticides, did you wear any protective clothing such as gloves or mask?

YES 01
NO 02
DK 888

11.	In the year before you became pregnant…<br><br>A. Did you personally apply pesticides at home?..	 <br>YES...................................... 01<br>NO..02<br>DK…………………………...888
12.	A. Did anyone other than you apply pesticides at your home?............................	YES...................................... 01<br>NO..02<br>DK…………………………...888

E. Pesticide Knowledge, Attitudes, Practices

Now I would like to ask you some general questions about human health and pesticides. Please answer as best you can according to your knowledge and opinions.

1. I believe that the following actions could be harmful to my fetus	Yes	No	DK
A. Smoking cigarettes	01	02	888
B. Eating fruits	01	02	888
C. Drinking alcohol	01	02	888
D. Spraying pesticides in the home	01	02	888
E. Spraying pesticides at work	01	02	888
F. Light exercise	01	02	888
G. Taking vitamins	01	02	888
H. Taking supplements	01	02	888

2. Exposure to pesticides can have an adverse effect or impact on human health

AGREE.................................. 01
DISAGREE.... **(GO TO 4)**...... 02
NOT SURE.... **(GO TO 4)**.... 888

3. If yes, do all the pesticides have the same adverse health effect on the human body?

YES 01
NO.. 02
NOT SURE.......................... 888

4. Pesticides can be harmful to the health of…	Yes	No	DK
A. The general population	01	02	888
B. The agricultural workers who apply them	01	02	888
C. Other agricultural workers	01	02	888
D. People who consume the crops	01	02	888
E. Farm residents	01	02	888
F. Residents of cities and communities near the farm	01	02	888

5. Which of the following are intake pathways for pesticides?	Yes	No	DK
A. Breathing in pesticides	01	02	888
B. Getting bit by a mosquito	01	02	888
C. Getting pesticides on the skin	01	02	888
D. Swallowing pesticides	01	02	888
E. Consuming foods from farms that use pesticides	01	02	888

6. It is necessary to read or understand the label of a pesticide bottle or container.

AGREE....................................01
DISAGREE………….................02
NOT SURE………….................888

7. Pesticide containers can be reused safely after cleaning.

AGREE....................................01
DISAGREE………….................02
NOT SURE………….................888

8. Which of the following can be effective in preventing pesticide exposure?	Yes	No	DK
A. Wearing full protective equipment when handling pesticides	01	02	888
B. Wearing gloves when handling pesticides	01	02	888
C. Washing fruits and vegetables before eating them	01	02	888
D. Covering mouth and nose with your hand while spraying pesticides	01	02	888
E. Washing hands in the stream after handling pesticides	01	02	888
F. Taking a bath immediately after spraying pesticides	01	02	888
G. Washing clothes worn at the farm separate from other clothes	01	02	888

9. According to your knowledge, the toxicity symptoms of pesticides can be which of the following?	Yes	No	DK
A. Headache	01	02	888
B. Watery eyes / sore eyes	01	02	888
C. Heart attack / stroke	01	02	888
D. Nausea / vomiting	01	02	888
E. Excessive salivation	01	02	888
F. Cough / cold / chest pain / breathlessness	01	02	888
G. Skin rash / skin irritation / itching	01	02	888
H. Abdominal pain / diarrhea	01	02	888
I. Muscle weakness / fatigue / body pain	01	02	888

10. Have you received training about pesticides?

YES.. 01
NO.......................**(GO TO 12)** 02
NOT SURE ....**(GO TO 12)** 888

11. If yes, please answer the following:

A. Where did you receive training? ____

B. When did you last receive training? ____

C. What topics were discussed in this training?

	Yes	No	DK
i. Pesticide poisoning	01	02	888
ii. Personal protective equipment	01	02	888
iii. Health effects of pesticides	01	02	888
iv. Proper spraying techniques	01	02	888
v. Other, specify ________________________	01	02	888

Do you agree or disagree with the following statements?	Agree	Disagree	Not sure
12. Pesticides protect people from pest-related diseases	01	02	888
13. Pesticides are poisonous	01	02	888
14. Pesticide hazard can cause death	01	02	888
15. You can smoke, drink, and eat during pesticide spraying	01	02	888
16. Using a large amount of pesticides for only a short time is not harmful to my health	01	02	888
17. Using a large amount of pesticides for only a short time is not harmful to the health of my fetus	01	02	888
18. Using a small amount of pesticides for a long time is not harmful to my health	01	02	888
19. Using a small amount of pesticides for a long time is not harmful to the health of my fetus	01	02	888
20. Adults are more resistant to pesticides than children	01	02	888
21. Adults are more resistant to pesticides than babies	01	02	888
22. If I eat and drink near areas where pesticides have been sprayed I will not be exposed to pesticides	01	02	888

23. If a pesticide is sold in the market it means it is safe no matter how or by whom it is used	01	02	888
24. A pesticide is effective only if its effect can be seen immediately after spraying	01	02	888
25. A pesticide is more effective if it is sprayed according to personal experience and not necessarily according to the recommended amount	01	02	888
26. Every person who uses a pesticide is responsible for its safe use	01	02	888
27. After using pesticides for a number of years, a person can develop an immunity to pesticides	01	02	888

28. Which of the following are potential health impacts of pesticides?	Yes	No	DK
A. Pesticide poisoning	01	02	888
B. Cancer	01	02	888
C. Obesity	01	02	888
D. Slower learning	01	02	888
E. Irritated skin	01	02	888
F. Coughing	01	02	888

29. I use pesticides in the home because…	Yes	No	DK
A. They protect my home and family from mosquitoes	01	02	888
B. They protect my home and family from other insects	01	02	888
C. They protect my home and family from rodents	01	02	888
D. They protect my home and family from termites	01	02	888
E. They protect my home and family from other pests	01	02	888
F. They protect my home and family from disease	01	02	888
G. They keep my home clean	01	02	888
H. A family member told me to	01	02	888
I. Following advice from a doctor, nurse, community leader, health volunteer, or government official	01	02	888
J. Other, Specify ______________________________	01	02	888

30. I use pesticides at work because…	Yes	No	DK
A. They kill insects that would harm the plants	01	02	888
B. They kill other pests that would harm the plants	01	02	888
C. They get rid of bacteria growing on the plants	01	02	888
D. They kill other unwanted plants	01	02	888
E. They make the plants grow taller	01	02	888
F. I am told to apply them	01	02	888
G. Other, Specify ______________________________	01	02	888

F. Demographics

1. How old are you? ____ ____ YEARS OLD

---

4. What is the last grade that you completed in school?

None, never attended school	01
P. 1-6 (primary)	02
M. 1-3 (junior high/high school)	03
M. 4-6 (high school/no diploma)	04
Diploma/technical school/equivalent	05
Some college	06
College graduate or more	07

---

5. Are you currently attending school?

Yes	01
No	02
DK	888
NR	999

---

7. How much money do you and other people who live in your home bring home each month? **(READ CATEGORIES)**

1,500 Baht or less	01
1,501 to 3,000 Baht	02
3,001 to 6,000 Baht	03
6,001 to 9,000 Baht	04
9,001 to 12,000 Baht	05
More than 12,000 Baht	06
DK	888
NR	999

---

9. Think about where you live, the food you eat, and the things you can afford to do and buy. Do you think you can afford these with your household income?

Yes, I can afford and have enough for saving	01
Yes, I can afford and not have enough for saving	02
No	03

---

10. Now I would like to ask you some questions about your ethnicity. What ethnic group best describes you?

Thai	01
Thai Yai	02
Burmese	03
Chinese	04
Other (SPECIFY) ______________	05
DK	888
NR	999

11. In what country were you born?

Thailand	01
Burma	02
China	03
Other (SPECIFY) ______________	04
DK	888
NR	999

G. Medical History

Now I would like to ask you questions about any vitamins that you take or have taken in the three months before pregnancy up until now.

19. In the 3 months before you became pregnant, did you take any prenatal or multivitamins?

Yes	01
No	02
DK	888
NR	999

20. Since you became pregnant, have you taken any prenatal or multivitamins?

Yes	01
No	02
DK	888
NR	999

H. Pregnancy History

1. Excluding this pregnancy, how many times have you been pregnant? (Probe: No matter what happened with the pregnancy)	____ ____ TIMES<br>DK.................. 888<br>NR 999
2. How many children do you have that are currently living with you?	____ ____ CHILDREN<br>DK.................. 888<br>NR 999

I. Paternal Demographics

1. What is your baby's father's ethnic background?	Thai .... 01<br>Thai Yai .... 02<br>Burmese .... 03<br>Chinese .... 04<br>Other (SPECIFY) _____________ 05<br>DK .... 888<br>NR 999
2. In what country was your baby's father born?	Thailand .... 01<br>Burma .... 02<br>China .... 03<br>Other (SPECIFY) _____________ 04<br>DK .... 888<br>NR 999
3. What is the last grade that your baby's father completed in school?	None, never attended school .... 01<br>P. 1-6 (primary) .... 02<br>M. 1-3 (junior high/high school) .... 03<br>M. 4-6 (high school/no diploma) ....04<br>Diploma/Technical school/equivalent .... 05<br>Some college ... .06<br>College graduate or more .... 07<br>DK .... 888<br>NR 999

J. Household Members

1. Do any of the people who live with you work in agriculture?	YES .... 01<br>NO .... 02<br>DK 888
4. Do any of these people (including yourself) usually wear their work shoes into your current home?	YES .... 01<br>NO .... 02<br>DK 888
6. Do any of these people wear their regular work clothes in your home for more than ½ hour before they change?	YES .... 01<br>NO .... 02<br>DK 888
8. Are these regular work clothes kept separately from other family clothes?	YES .... 01<br>NO .... 02<br>DK 888
12. Are these work clothes mixed with the family wash or washed separately?	Mixed with family wash .... 01<br>Washed separately .... 02<br>DK 888
13. Does anyone store containers or bags of pesticides from work in or around the home you live in now?	YES .... 01<br>NO .... 02<br>DK 888

K. Pets

I would like to know about any pets that have lived inside your home since you became pregnant. Please include any dogs, cats, birds, or other furry pets that belong to you or to anyone who lives inside your home, including people who are not related to you.

2. Since you became pregnant, have you personally applied flea or tick shampoo, dips or powders on any of your pets?	YES .... 01<br>NO ... **(GO TO Q. 4)** .... 02<br>DK ... **(GO TO Q. 4)** .. 888
3. Did you wear gloves when you used these products?	YES .... 01<br>NO 02

M. Personal Habits Information

Now I would like to ask you some questions about your smoking habits.

6. In the three months before you became pregnant, did you smoke any cigarettes?	YES .... 01<br>NO .... **(GO TO Q. 8)** .... 02<br>DK .... **(GO TO Q. 8)** 888
8. Since you became pregnant, have you smoked any cigarettes?	YES .... 01<br>NO .... **(GO TO Q. 10)** .... 02<br>DK .... **(GO TO Q. 10)** 888

N. Other Exposures and Concerns

4. Do you know of any effects that pesticides or other environmental exposures in Fang District may have had on you or your family? Please mention any and all problems that come to mind.

YES 01
NO 02
DK 888

A. Please explain: ___________________________

____

____

Z. Additional Questions

1. How long have you lived in Fang? ____ ____ YEARS ____ ____ MONTHS
(99: Since I was born)

2. Is your current house near an agricultural area?
   - I live in an agricultural area/orchard/farm 01
   - I live near an agricultural area (<500m)................. 02
   - I don't live near an agricultural area (>500m)........ 03
   - DK.. 888
   - NR 999

3. What Medicare benefits have you used for antenatal care for this pregnancy?
   - Gold health card 01
   - Health insurance card for foreigner 02
   - Medicare benefit for officer 03
   - Social Security... 04
   - None (paid by myself) 05
   - Other, (SPECIFY)________________ 06
   - DK 888
   - NR 999

4. What month of pregnancy was your first visit to ANC? ____ ____ MONTHS
(888: DK)

5. During this pregnancy, how many times have you visited ANC? ____ ____ TMES
(888: DK)

6. During this pregnancy, have you visited ANC for every regular appointment?
   - Yes, on time every appointment 01
   - Yes, but not on time every appointment 02
   - No, because ______________________ .. 03

7. Currently, do you still work?
   - YES **(GO TO Q. 8)**.. 01
   - NO.. **(GO TO Q. 7A)**.. 02

7A. One year ago, did you work?
   - YES **(GO TO Q. 7B)**.. 01

7B. When did you stop working?	Before I knew that I was pregnant .... 01<br>After I knew that I was pregnant .... 02<br>(at ___ ___ month of pregnancy because<br>___________________________________<br>___________________________________)
8. What is your employment status?	I have my own business/orchard/farm................... 01<br>I am an employee .... 02<br>Other, Specify______________________________ 03
8A. When do you plan to stop working?	____ ____ MONTHS<br>When doctor orders or I can't work anymore...... 77<br>I will work up until delivery .... 99<br>DK.. 888
9. After delivery, do you plan to work?	YES **(GO TO Q. 9A)**.. 01<br>NO... **(GO TO Q. 10)**.. 02<br>DK .. **(GO TO Q. 9A)**.. 02
9A. If you plan to work after delivery, does your work involve agriculture?	YES .... 01<br>NO 02
9B. When do you plan to start working again?	Within 3 months after delivery .... 01<br>Within 6 months after delivery .... 02<br>After 6 months after delivery .... 03<br>DK 888
10. Do you plan to move from Fang?	YES.**(GO TO Q. 10A)**. 01<br>NO... **(GO TO Q. 11)**.. 02<br>DK.....**(GO TO Q. 11)**. 02

10A. When do you plan to move from Fang?	Within 3 months after delivery .... 01<br>Within 6 months after delivery .... 02<br>After 6 months after delivery .... 03<br>DK .... 888<br>Reason ______________________________<br>___________________________________
11. Will you breast feed your baby by yourself?	Yes............ **(GO TO Q. 11A)** .... 01<br>No.. 02<br>DK .... 888<br>Reason for No or DK __________________<br>___________________________________<br>___________________________________
11A. How long will you breast feed your baby?	3 months after delivery .... 01<br>6 months after delivery .... 02<br>1 year after delivery .... 03<br>Over 1 year after delivery .... 04<br>DK 888

INTERVIEWER REMARKS

PLEASE ANSWER THE FOLLOWING QUESTIONS IMMEDIATELY AFTER THE INTERVIEW.

1. Overall, the respondent's cooperation was:

Excellent..............................01
Good....................................02
Fair......................................03
Poor.....................................04

Table A1. Knowledge score calculation.

**Knowledge Questions**	**Response Considered Correct**	**Source**
I believe that the following actions could be harmful to my fetus:		
Smoking cigarettes	Yes	[1]
Eating fruits	No	[2]
Drinking alcohol	Yes	[3]
Spraying pesticides in the home	Yes	[4]
Spraying pesticides at work	Yes	[5]
Light exercise	No	[6]
Taking vitamins	No	[7]
Taking supplements	No	[7]
Exposure to pesticides can have an adverse effect or impact on human health	Agree	[8]
Do all the pesticides have the same adverse health effect on the human body?	No	[8]
Pesticides can be harmful to the health of:		
The general population	Yes	[9]
The agricultural workers who apply them	Yes	[10]
Other agricultural workers	Yes	[11]
People who consume the crops	Yes	[12]
Farm residents	Yes	[11]
Residents of cities and communities near the farm	Yes	[11]
Which of the following are intake pathways for pesticides?		
Breathing in pesticides	Yes	[12]
Getting bit by a mosquito	No	
Getting pesticides on the skin	Yes	[12]
Swallowing pesticides	Yes	[12]
Consuming foods from farms that use pesticides	Yes	[12]
Pesticide containers can be reused safely after cleaning	Disagree	[11]
Which of the following can be effective in preventing pesticide exposure?		
Wearing full protective equipment when handling pesticides	Yes	[13]
Wearing gloves when handling pesticides	Yes	[13]
Washing fruits and vegetables before eating them	Yes	[11]
Covering mouth and nose with your hand while spraying pesticides	No	[14]
Washing hands in the stream after handling pesticides	Yes	[15]
Taking a bath immediately after spraying pesticides	Yes	[15]
Washing clothes worn at the farm separate from other clothes	Yes	[11]
According to your knowledge, the toxicity symptoms of pesticides can be which of the following?		
Headache	Yes	[11]
Watery eyes / sore eyes	Yes	[8]
Heart attack / stroke	Yes	[11]
Nausea / vomiting	Yes	[11]
Excessive salivation	Yes	[11]
Cough / cold / chest pain / breathlessness	Yes	[8]
Skin rash / skin irritation / itching	Yes	[11]
Abdominal pain / diarrhea	Yes	[11]
Muscle weakness / fatigue / body pain	Yes	[11]

Table A1. *Cont.*

**Knowledge Questions**	**Response Considered Correct**	**Source**
Pesticides protect people from pest-related diseases	Agree	[16]
Pesticides are poisonous	Agree	[11]
Pesticide hazard can cause death	Agree	[17]
You can smoke, drink, and eat during pesticide spraying	Disagree	[11]
If I eat and drink near areas where pesticides have been sprayed I will not be exposed to pesticides	Disagree	[18]
Which of the following are potential health impacts of pesticides?		
Pesticide poisoning	Yes	[19]
Cancer	Yes	[20]
Obesity	No *	[21]
Slower learning	Yes	[22]
Irritated skin	Yes	[8]
Coughing	Yes	[19]

* Although some prospective epidemiological studies have reported an association between prenatal exposure to dichlorodiphenyl-dichloroethane (DDE) and measures of obesity in childhood, the toxicological evidence is limited and there is a lack of general consensus regarding the potential relationship between pesticide exposure and obesity.

Table A2. Personal susceptibility attitude score calculation.

**Attitudes on personal susceptibility to health effects from pesticides ***	**Response indicating higher belief in susceptibility ^**
Using a large amount of pesticides for only a short time is not harmful to my health	Disagree
Using a small amount of pesticides for a long time is not harmful to my health	Disagree

* Score ranges from 0–4 with higher scores indicating a higher belief in personal susceptibility to health effects from pesticides.

^ These responses were awarded 2 points, while the opposite response was awarded 0 points. Responses of “not sure” or “don’t know” (indicating beliefs in between the extremes) were awarded 1 point.

Table A3. Child susceptibility attitude score calculation.

**Attitudes on future or current children's susceptibility to health effects from pesticides** *	**Response indicating higher belief in susceptibility** ^
Using a large amount of pesticides for only a short time is not harmful to the health of my fetus	Disagree
Using a small amount of pesticides for a long time is not harmful to the health of my fetus	Disagree
Adults are more resistant to pesticides than children	Agree
Adults are more resistant to pesticides than babies	Agree

* Score ranges from 0–8 with higher scores indicating a higher belief in the participant's child's susceptibility to health effects from pesticides.

^ These responses were awarded 2 points, while the opposite response was awarded 0 points. Responses of "not sure" or "don't know" (indicating beliefs in between the extremes) were awarded 1 point.

Table A4. Responsibility attitude score calculation.

**Attitudes on responsibility for safe use based on Sam *et al.* [23]** *	**Response indicating acceptance of responsibility** ^
It is necessary to read or understand the label of a pesticide bottle or container	Agree
If a pesticide is sold in the market it means it is safe no matter how or by whom it is used	Disagree
A pesticide is effective only if its effect can be seen immediately after spraying	Disagree
A pesticide is more effective if it is sprayed according to personal experience and not necessarily according to the recommended amount	Disagree
Every person who uses a pesticide is responsible for its safe use	Agree
After using pesticides for a number of years, a person can develop an immunity to pesticides	Disagree

* Score ranges from 0–12 with higher scores indicating a higher acceptance of personal responsibility for the safe use of pesticides.

^ These responses were awarded 2 points, while the opposite response was awarded 0 points. Responses of "not sure" or "don't know" (indicating beliefs in between the extremes) were awarded 1 point.

Table A5. Usefulness attitude score calculation.

**Attitudes on the usefulness of pesticides** *	**Does an affirmative response indicate belief in pesticide usefulness?** ^
I use pesticides in the home because:	
They protect my home and family from mosquitoes	Yes
They protect my home and family from other insects	Yes
They protect my home and family from rodents	Yes
They protect my home and family from termites	Yes
They protect my home and family from other pests	Yes
They protect my home and family from disease	Yes
They keep my home clean	Yes
A family member told me to	No
Following advice from a doctor, nurse, community leader, health volunteer, or government official	No
Other	Yes
I use pesticides at work because:	
They kill insects that would harm the plants	Yes
They kill other pests that would harm the plants	Yes
They get rid of bacteria growing on the plants	No
They kill other unwanted plants	Yes
They make the plants grow taller	Yes
I am told to apply them	No
Other	Yes

* Score ranges from 0–13 with higher scores indicating a higher belief in the usefulness of pesticides (scored as missing if the participant did not personally apply pesticides).

^ Affirmative responses to questions with a "yes" in this category were awarded 1 point each, while all other responses were awarded 0 points.

Table A6. Risky behaviors defined by Goldman *et al.* [24].

**Risky behaviors defined by Goldman *et al.* [24]**	**Corresponding KAP survey question(s)**
Sometimes or never washing hands in the field before smoking or eating	A20
Not bathing immediately after work	A21
Not wearing adequate clothing to protect against pesticide exposure (long-sleeved shirt, something to cover the head, and gloves)	A18, D5, K3
Storing or washing farm-worker clothes together with family clothes	J8, J12
Cleaning the house less than a few times per week	C2
Eating fruits and vegetables directly from the field	B11A
Household member(s)wearing work shoes from the field into the home	J4
Household member(s)wearing work clothes from the field into the home for more than 30 minutes	J6

Table A7. Risky behaviors at work score calculation.

Risky behaviors at work *	Corresponding KAP survey question(s)
Sometimes or never washing hands in the field before smoking or eating	A20
Not bathing immediately after work	A21
Not wearing adequate clothing to protect against pesticide exposure (long-sleeved shirt, something to cover the head, and gloves)	A18

* These scores were only calculated for participants who worked in agriculture while pregnant.

Table A8. Risky behaviors at home score calculation.

Risky behaviors at home	Corresponding KAP survey question(s)
Not wearing personal protective equipment when using pesticides in the home	D5, K3
Storing or washing farm-worker clothes together with family clothes *	J8, J12
Cleaning the house less than a few times per week	C2
Eating fruits and vegetables directly from the field	B11A
Household member(s) wearing work shoes from the field into the home *	J4
Household member(s) wearing work clothes from the field into the home for more than 30 minutes *	J6
Household member(s) storing pesticides from work in or around the home	J13

* Only considered risky when participants had household members who worked in agriculture.

References

1. Cotton, P. Smoking cigarettes may do developing fetus more harm than ingesting cocaine, some experts say. *J. Am. Med. Assoc.* **1994**, *271*, 576–577.
2. Ortega, R.M. Dietary guidelines for pregnant women. *Public Health Nutr.* **2001**, *4*, 1343–1346.
3. Eustace, L.W.; Kang, D-H.; Coombs, D. Fetal alcohol syndrome: A growing concern for health care professionals. *JOGNN* **2002**, *32*, 215–221.
4. Turner, M.C.; Wigle, D.T.; Krewski, D. Residential pesticides and childhood leukemia: A systematic review and meta-analysis. *Environ. Health Perspect.* **2010**, *118*, 33–41.
5. Wigle, D.T.; Turner, M.C.; Krewski, D. A systematic review and meta-analysis of childhood leukemia and parental occupational pesticide exposure. *Environ. Health Perspect.* **2009**, *117*, 1505–1513.
6. Artal, R.; O'Toole, M. Guidelines of the American College of Obstetricians and Gynecologists for exercise during pregnancy and the postpartum period. *Brit. J. Sport. Med.* **2003**, *37*, 6–12.
7. Haider, B.A.; Bhutta, Z. Multiple-micronutrient supplementation for women during pregnancy. *Cochrane Database Syst. Rev.* **2006**, *4*, doi:10.1002/14651858.CD004905.

8. U.S. Environmental Protection Agency (EPA). *Recognition and Management of Pesticide Poisonings*; fifth ed.; EPA/735/R-98/003; EPA: Washington, DC, USA, **1999**.
9. National Cancer Institute (NCI). *Cancer Trends Progress Report—2009/2010 Update*. National Institutes of Health: Bethesda, MD, USA, 2010.
10. Das, R.; Steege, A.; Baron, S.; Beckman, J.; Harrison, R. Pesticide-related illness among migrant farm workers in the United States. *Int. J. Occup. Env. Heal.* **2001**, *7*, 303–312.
11. U.S. Environmental Protection Agency (EPA). *Citizen's Guide to Pest Control and Pesticide Safety*; EPA/735/K/04/002; EPA: Washington, DC, USA, 2005.
12. McKone, T.E.; Castorina, R.; Harnly, M.E.; Kuwabara, Y.; Eskenazi, B.; Bradman, A. Merging models and biomonitoring data to characterize sources and pathways of human exposure to organophosphorus pesticides in the Salinas Valley of California. *Environ. Sci. Technol.* **2007**, *41*, 3233–3240.
13. Keifer, M.C. Effectiveness of interventions in reducing pesticide overexposure and poisonings. *Am. J. Prev. Med.* **2000**, *18*, 80–89.
14. Palis, F.G.; Flor, R.J.; Warburton, H.; Hossain, M. Our farmers at risk: behaviour and belief system in pesticide safety. *J. Public Health* **2006**, *28*, 43–48.
15. Salvatore, A.L.; Bradman, A.; Castorina, R.; Camacho, J.; Lopez, J.; Barr, D.B.; Snyder, J.; Jewell, N.P.; Eskenazi, B. Occupational behaviors and farmworkers' pesticide exposure: Findings from a study in Monterey County, California. *Am. J. Ind. Med.* **2008**, *51*, 782–794.
16. Guillet, P.; Alnwick, D.; Cham, M.K.; Neira, M.; Zaim, M.; Heymann, D.; Mukelabai, K. Long-lasting treated mosquito nets: A breakthrough in malaria prevention. *Bull. World Health Organ.* **2001**, *79*, 998.
17. Casey, P. Deaths from pesticide poisoning in England and Wales: 1945–1989. *Hum. Exp. Toxicol.* **1994**, *13*, 95–101.
18. Fenske, R.A.; Black, K.G.; Elkner, K.P.; Lee, C.L.; Methner, M.M.; Soto, R. Potential exposure and health risks of infants following indoor residential pesticide applications. *Am. J. Public Health* **1990**, *80*, 689–693.
19. Eskenazi, B.; Bradman, A.; Castorina, R. Exposures of children to organophosphate pesticides and their potential adverse health effects. *Environ. Health Perspect.* **1999**, *107*, 409–419.
20. Vinson, F.; Merhi, M.; Baldi, I.; Raynal, H.; Gamet-Payrastre, L. Exposure to pesticides and risk of childhood cancer: A meta-analysis of recent epidemiological studies. *Occup. Environ. Med.* **2011**, *68*, 694–702.
21. La Merrill, M.; Birnbaum, L.S. Childhood obesity and environmental chemicals. *Mt. Sinai J. Med.* **2011**, *78*, 22–48.
22. Kofman, O.; Berger, A.; Massarwa, A.; Friedman, A.; Jaffar, A.A. Motor inhibition and learning impairments in school-aged children following exposure to organophosphate pesticides in infancy. *Pediatr. Res.* **2006**, *60*, 88–92.
23. Sam, K.G.; Andrade, H.H.; Pradhan, L.; Pradhan, A.; Sones, S.J.; Rao, P.G.; Sudhakar, C. Effectiveness of an educational program to promote pesticide safety among pesticide handlers of South India. *Int. Arch. Occ. Environ. Hea.* **2008**, *81*, 787–795.

24. Goldman, L.; Eskenazi, B.; Bradman, A.; Jewell, N.P. Risk behaviors for pesticide exposure among pregnant women living in farmworker households in Salinas, California. *Am. J. Ind. Med.* **2004**, *45*, 491–499.

© 2012 by the authors; licensee MDPI, Basel, Switzerland. This article is an open access article distributed under the terms and conditions of the Creative Commons Attribution license (http://creativecommons.org/licenses/by/3.0/).
